# Thin Skin in Rhinoplasty: Considerations for Camouflaging Dorsal Nasal Irregularities

**DOI:** 10.7759/cureus.66595

**Published:** 2024-08-10

**Authors:** Reda M Daoud, Abdulrahman A Alelyani, Basel H Bakhamees, Ruyuf F Almutairi, Naif AlSufyani, Ahmed Y Ayoub, Abdulaziz M Alshehri, Hatoun A Alali, Bayan W Basri, Rema M Alhazmi, Alghaydaa Aldoughan, Juri Alghofaili, Ebtehal M Alhayyan, Bassam H Awaji

**Affiliations:** 1 Otolaryngology (ENT), King Salman Armed Forces Hospital in Nortwestren Region, Tabuk, SAU; 2 Medicine, University of Tabuk, Tabuk, SAU; 3 Medicine, King Abdulaziz University, Jeddah, SAU; 4 Medicine and Surgery, Taif University, Taif, SAU; 5 Medicine, Jazan University, Gizan, SAU; 6 Medicine, King Khalid University, Abha, SAU; 7 Medicine and Surgery, University of Tabuk, Tabuk, SAU; 8 Hospital Medicine, Manchester Royal Infirmary, Manchester, GBR; 9 Otorhinolaryngology, Taibah University, AlMadinah AlMunawwarah, SAU; 10 Medicine, King Faisal University, Al-Ahsa, SAU; 11 Medicine, Qassim University, Buraydah, SAU; 12 Critical Care, Abqaiq General Hospital, Qatif, SAU; 13 Otolaryngology (ENT), King Fahad Specialist Hospital, Riyadh, SAU

**Keywords:** laser-assisted rhinoplasty, fascia lata grafts, graft integration, dorsal nasal irregularity, thin nasal skin, rhinoplasty

## Abstract

Thin skin presents a challenge for achieving optimal aesthetic outcomes and minimizing complications. The review analyzes various materials and techniques employed to achieve this goal. A comprehensive electronic search was conducted across various medical databases, retrieved 965 studies, from which 15 studies were eligible for inclusion in this review with a total number of 679 patients with thin nasal skin. Techniques that promote graft integration, minimize resorption, and provide a smooth dorsal contour are crucial for thin-skinned patients. Diced cartilage with PRP, fascia lata grafts, and laser-assisted rhinoplasty appear to be particularly effective based on the available evidence. Platelet-rich fibrin (PRF) appears to play a role in some techniques by enhancing healing and tissue regeneration. Natural materials, like fascia lata and ligamentous grafts, offer potential benefits but require further exploration. Fat grafting techniques show promise but necessitate more research. This review provides a comprehensive overview of various techniques for addressing dorsal irregularities in rhinoplasty for patients with thin skin. Surgeons can utilize this information to select the most appropriate approach for achieving optimal aesthetic outcomes while minimizing complications.

## Introduction and background

Rhinoplasty, also known as nose reshaping, is one of the most commonly performed cosmetic procedures worldwide [1]. Achieving optimal aesthetic and functional outcomes in rhinoplasty relies on meticulous surgical technique and careful consideration of various anatomical factors. One such crucial factor is nasal skin thickness [2].

The thin nasal skin is a delicate tissue that offers minimal camouflage for the underlying skeletal framework. This can lead to several potential complications, including visibility of underlying structures such as bony irregularities and cartilage contours more apparent, potentially affecting the aesthetic outcome. Furthermore, achieving a sculpted and defined nasal tip can be challenging with thin skin, as aggressive maneuvers might lead to irregularities or shine-through [3]. Thin skin might be more susceptible to complications like wound healing problems, skin necrosis, and scar formation [4].

Understanding the specific considerations for rhinoplasty in patients with thin skin is critical for surgeons to optimize outcomes, minimize complications, and achieve patient satisfaction [1]. This systematic review provides a comprehensive analysis of the current evidence regarding considerations for thin-skinned patients undergoing rhinoplasty to camouflage or correct dorsal nasal deformities to inform evidence-based surgical decision-making.

## Review

Methods

This systematic review followed the Preferred Reporting Items for Systematic Reviews and Meta-Analyses (PRISMA) guidelines [5]. 

Search strategy

A comprehensive electronic search was conducted across various medical databases, including PubMed, MEDLINE, Embase, and Cochrane Central Register of Controlled Trials (CENTRAL). The search strategy employed relevant keywords and MeSH terms related to rhinoplasty, thin skin, and surgical considerations. Boolean operators (AND, OR, NOT) were used to combine search terms and refine the results. The search was performed on articles published in English from 2000 until June 28, 2024 (Appendix A).

Inclusion and exclusion criteria

Study design included clinical trials, observational studies (cohort studies, case-control studies), and case series reporting on rhinoplasty outcomes in patients with thin nasal skin. The participants included adults undergoing primary or revision rhinoplasty. Surgical techniques were specifically tailored for rhinoplasty in patients with thin skin. Aesthetic and functional outcomes of rhinoplasty included patient satisfaction, complication rates, and long-term results.

Excluded studies were either not published in English, were reviews, editorials, or letters to the editor, did not address rhinoplasty considerations for thin skin, or involved animal or cadaveric studies.

Selection of studies

Two independent reviewers screened titles and abstracts of all retrieved citations to identify potentially relevant studies. Full-text articles of studies meeting the inclusion criteria were retrieved and further assessed for eligibility by the same reviewers. Disagreements were resolved through discussion or by consulting a third reviewer.

Data extraction

A standardized data extraction form was developed to record relevant information from the included studies. The data extracted included study characteristics (author, year, study design), patient demographics (age, sex), surgical technique details, and outcome measures (aesthetic outcomes, complications, patient satisfaction).

Data synthesis

**Figure 1:**
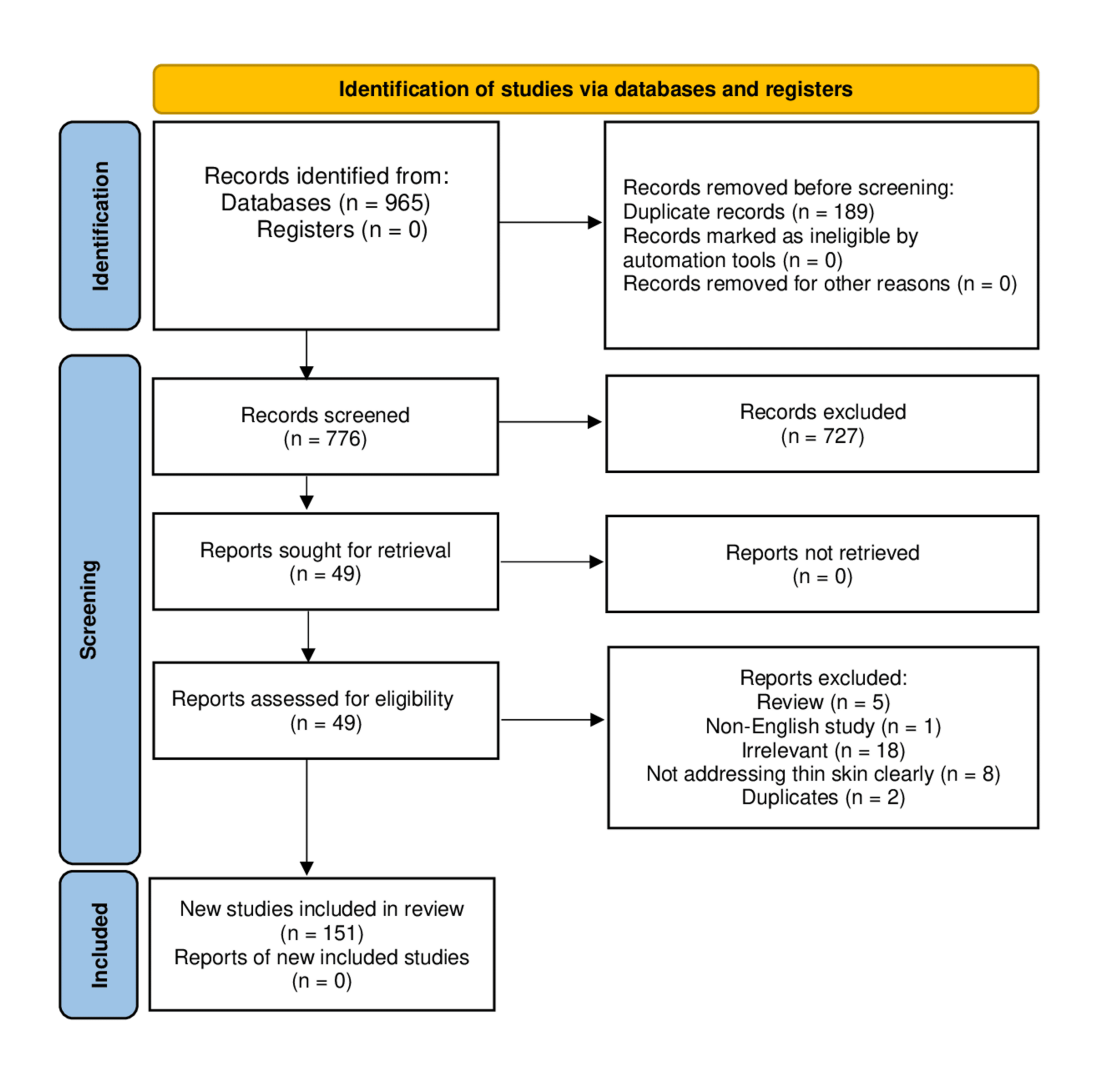
PRISMA flow chart of the included studies.

The initial search resulted in 965 studies. After the removal of duplicates and excluded studies, 15 studies with a total number of 890 patients (of which 679 patients had nasal thin skin) were eligible for this review (Figure 1) [6]. Extracted data were tabulated (Table 1) and summarized narratively.

 

Level of evidence

The level of evidence of the included studies was made using the Grading of Recommendations Assessment, Development, and Evaluation (GRADE) approach which ranks evidence from high (RCTs with low risk of bias) to very low (observational studies with high risk of bias) [7].

 

**Table 1:** Summary table of the included studies M = male, n = number, NM = not mentioned, NA = not applicable.

Author, year	Country	study design	Number of patients	Age range(average)	Sex (m%)	Surgical techniques	Outcomes	Adverse effects	patients’ satisfaction	Treatment follow up (in months)	level of evidence	
Santos et al., 2020 [8]	Portugal	prospective study	200 (84 thin skin)	average 35.44 y	35%	Diced Cartilage (DC) Vs Shaved cartilage gel Plus Platelet-Rich Fibrin (SC+PRF)	SC+PRF resulted in better long-term aesthetic outcomes than DC, especially for patients with thin skin (p=0.001). SC+PRF achieved better outcomes regardless of the surgical approach (closed vs open rhinoplasty). Patients with SC+PRF had fewer postoperative irregularities on the nasal dorsum requiring correction compared to DC. No revision rhinoplasty was needed in the SC+PRF group, while 10 patients in the DC group required revision.	No major side effects were reported	Both groups showed improvement in aesthetic scores over time.	after 3 and 12	II	
Hanci et al., 2019 [9]	Turkey	Case series	52	22-40 (26.2 y)	61.53%	diced cartilage mixed with NCH gel	No irregularities, displacement, or absorption of the camouflage material observed. Patients satisfied with the aesthetic results. No adhesions at the osteotomy lines. No revision rhinoplasties needed.	No complications reported.	The study mentions patient satisfaction but doesn't provide specific data.	12	IV	
Barone et al., 2020 [10]	Italy	RCT	101	18–65 (38.5 y)	36.63%	diced cartilage Vs lipofilling Vs temporal fascia VS No camouflage	Patients in the diced cartilage group (Group 1) reported the highest satisfaction scores on the FACE-Q test (p < 0.01). Group 1 also required the fewest secondary procedures. Both surgeons rated the aesthetic outcomes of the diced cartilage (Group 1) and fascia graft (Group 3) techniques as superior (p < 0.01).		Diced cartilage camouflage resulted in the highest satisfaction scores for both patients and surgeons.	30	I	
Zheng et al., 2023 [11]	China	prospective (no controls)	52	average 31.01±9.19 y	11.53%	Perichondrial edge graft for dorsal augmentation	Patients reported significant improvement in aesthetics and function based on the ROE scale (p < 0.001). Increased satisfaction with the nasal dorsum was reported on the VAS scale (pre-op: 2.45, post-op: 8.55).	minimal complications as: 1 case of fat liquefaction (scarring) at the donor site. 2 cases required revision surgery due to graft warping.	Patients reported high satisfaction with the outcome based on standardized scales.	6 to 20	IV	
												Karaaltın et al., 2007 [12]	Turkey	prospective (no controls)	63	18 -43(28.7 y)	41.26%	Fascia Lata graft for dorsal camouflage in rhinoplasty	Questionnaire results showed satisfaction with the surgery and minimal donor site concerns. No visible irregularities were observed on the nasal dorsum post-surgery.	one patient reported scar and pain at the thigh donor site.	that all patients were satisfied with the cosmetic results	14-26	IV	
Cardenas et al., 2007 [13]	Colombia	prospective (no controls)	78	14-56 (15 y)	8.97%	lipoinjection of microfat grafts	High patient satisfaction with the procedure. One patient reported dissatisfaction unrelated to lipoinjection.	No major side effects reported at the injection sites or fat harvest area.	77 patients satisfied with the results.	1 to 36	IV													
												Lozada et al., 2020 [14]	USA	prospective (no controls)	49	NM	NM	supracrural ligament graft	The supracrural ligament graft was a safe, simple, and effective camouflage graft for irregularities in rhinoplasty	No reported complications	NM	NM	IV	
Mohebbi et al., 2016 [15]	Iran	RCT	24	22–48(31.25±6.93 y)	16.70%	Acellular dermis (ACD) Vs fascia lata	Fascia lata grafts resulted in less dorsal irregularity as detected by feel (palpation) at 12 months compared to ACD grafts. ACD grafts showed a higher absorption rate than fascia lata grafts at 6 months, but this difference disappeared by 12 months.	No major complications were reported in either group.	Patients were more satisfied with the results of fascia lata grafts.	3,6 and 12	I	
			18	25-55(34.94±9.04 y)	16.70%							
Bertossi et al., 2017 [16]	Italy	prospective (no controls)	57	31- 55y	45.61%	cartilage grafts in conjunction with collagen membrane.	The bilayered graft may lead to superior aesthetic outcomes with a balanced nasal tip and a fuller dorsum. The technique avoids altering the thickness and texture of the overlying skin.	NM	Not assessed directly	6 to 12	IV	
Ozturan et al., 2018 [17]	Turkey	prospective (no controls)	11 (8 thin skin)	NM	54.50%	dermocartilaginous ligament as a flap to cover the nasal dorsum	There were no visible irregularities on the nasal dorsum post-operatively.	NM	all patients were satisfied with the aesthetic outcome.	6 to 37	IV	
Bertossi et al., 2021 [18]	Italy	Cohort	25	18–51(27y)	52%	classic rhinoplasty Vs laser-assisted rhinoplasty	Laser-assisted rhinoplasty was feasible and safe. Laser group had a slightly longer operative time. Laser group showed less bleeding and faster edema resolution. Independent analysis showed a lower rate of residual defects in the laser group.	No major complications were observed in either group.	Patient satisfaction was higher in the laser group.	24–36	II	
		Cohort	25	18–44(23y)	56%	laser-assisted rhinoplasty						
												Kovacevic et al., 2021 [19]	Germany	Case series	107	NA	NA	Diced macrofat transfer bonded with PRF to the nasal dorsum	Good skin quality, no signs of shrinkage, marked scarring, or color changes, positive skin mobility test in all patients, fat survival confirmed by imaging	No perioperative complications or wound-healing issues	Not explicitly mentioned	14	IV	
												Storck et al., 2024 [20]	Germany	Case series	21	NA	NA	Autologous fat transfer with platelet-rich fibrin (PRF)	Improved skin quality, concealed irregularities, optimized skin mobility	Not reported	Not assessed directly	12	IV	
Niechajev et al., 2011 [21]	Sweden	Prospective case series	44	18 -47 (27 y)	34%	Skoog rhinoplasty	The study found favorable outcomes in all cases. The Skoog technique effectively created a smooth nasal bridge and prevented irregularities that can occur with traditional reduction rhinoplasty. Additionally, it served as a useful rescue technique when too much dorsum was removed during surgery.	Very thin grafts sometimes resulted in visible and palpable edges, requiring secondary shaving or filing in four patients.	Not assessed directly but mentioned desired aesthetic outcomes based on patient requests	12	IV	
Baptista et al., 2013 [22]	France	Prospective case series	20	average 53y	only women	lipofilling to correct sequelae following rhinoplasty surgery.	Eighteen patients achieved satisfactory results after one procedure. Two patients required a second lipofilling session. Complications were minimal, with some patients experiencing bruising and swelling after the Coleman technique procedure. The microinjection technique resulted in less bruising and swelling.	Bruising and swelling due to the Coleman technique for fat harvesting.	Not assessed directly but mentioned desired aesthetic outcomes based on patient requests	18-24	IV	

 

Results

Santos et al. [8] conducted a prospective study comparing two different techniques for dorsal camouflage after rhinoplasty: diced cartilage (DC) and shaved cartilage plus platelet-rich fibrin (SC+PRF). The study involved 200 Caucasian Mediterranean patients undergoing primary reduction rhinoplasty. The patients had a mean age of 35.44 years undergoing primary reduction rhinoplasty (132 in the DC group, 68 in the SC+PRF group). Aesthetic outcomes were assessed using the Utrecht Questionnaire (UQ) with a visual analog scale (VAS) before and after surgery (3 and 12 months). Regarding skin type, there were 84 (42.0%) thin-skinned, 87 (43.5%) intermediate-skinned, and 29 (14.5%) thick-skinned patients.

In the DC method, the cartilage was cut into small pieces and used as a paste for dorsal camouflage. While in the SC+PRF technique; the cartilage is shaved into thin pieces and mixed with a fibrin clot derived from the patient's own blood.

The key finding is that according to VAS scores, SC+PRF (pre-operative: 3.85 ± 0.17, post-operative: 8.19 ±0.12) resulted in significantly better aesthetic outcomes compared to DC (pre-operative: 3.78±0.10, post-operative: 7.79 ±0.09), particularly at 12 months post-surgery (p=0.004). This improvement was most notable in patients with thin skin (p = 0.001). Additionally, the SC+PRF group experienced no postoperative nasal dorsum irregularities, whereas 10 patients showed some irregularities requiring correction occurred in the DC group.

Overall, the study suggests that SC+PRF offers advantages over DC in dorsal camouflage rhinoplasty, especially for patients with thin skin or those requiring more extensive camouflage. The benefits of SC+PRF were statistically significant and independent of the surgical approach (open or closed) (p = 0.017, p = 0.046), respectively.

Level of Evidence II: A Well-Designed Prospective Comparative Trial

Hanci et al. explored a new technique for covering grafts used in rhinoplasty (dorsal camouflage) on thin-skinned patients [9]. A total of 52 patients (29 females and 23 males) underwent primary open approach rhinoplasty with nasal dorsum enhancement using DC, NCH gel, and blood mixture. Their ages ranged between 22 and 40 years (mean age, 26.2 years).

In this technique, cartilage grafts were harvested from the septum, tragus, and concha of the ear or rib. The DC was mixed with 1 cc of NCH gel and peripheral blood to form a paste-like consistency, and the mixture was applied to the nasal dorsum using a dorsal retractor. No irregularities, displacement, or absorption of the camouflage material were observed after one year. There were no complications, and all patients were satisfied with the aesthetic results.

The authors reported that this method is effective, simple, and timesaving, and reduces adhesions at the osteotomy lines. Overall, this pilot study suggests that DC with NCH gel and blood might be a promising technique for dorsal camouflage in rhinoplasty, particularly for thin-skinned patients. However, due to the study design limitations (small sample size, no control group, relatively short follow-up), more robust studies are needed for conclusive evidence.

Level of Evidence IV: Case Series With No Control Group

Barone et al. conducted an RCT comparing different techniques for covering grafts used in rhinoplasty (dorsal camouflage) on patients with thin skin [10]. One hundred one patients (64 females and 37 males) with confirmed thin skin underwent primary rhinoplasty (both functional and cosmetic). The average patient’s age was 38.5 years.

Patients were randomly assigned to one of four groups, Group 1 (n=27): DC Camouflage - Cartilage pieces used to cover the dorsum. Group 2 (n=25): Lipofilling Camouflage - Fat grafting used for camouflage. Group 3 (n=24): Temporal Fascia Graft Camouflage - Fascia tissue from the temple used for covering. Group 4 (n=25): No Camouflage (Control Group). All patients were followed up for an average of 2.5 years (provides a good assessment of mid-term outcomes). Patient satisfaction was measured using the FACE-Q rhinoplasty module and satisfaction with appearance was rated by two plastic surgeons.

The results of this study showed that patients in group 1 (DC) showed the greatest improvement in FACE-Q scores after surgery compared to other groups (p < 0.01). Patient satisfaction scores were also highest in group 1, as rated by both patients and surgeons (p < 0.01). Groups 2 and 4 (lipofilling and no camouflage) required more secondary procedures compared to groups 1 and 3 (DC and temporal fascia) (p < 0.01).

This study suggests that DC grafts are the most effective technique for dorsal camouflage in rhinoplasty patients with thin skin. It leads to higher patient satisfaction, better long-term aesthetics, and requires fewer revision surgeries compared to other methods evaluated.

Level of Evidence I: A Randomized Controlled Trial With Strong Evidence

Zheng et al. described a retrospective analysis of open augmentation rhinoplasty with modified perichondrium on dorsal onlay graft in patients with thin skin [11]. 52 thin-skinned Chinese patients (age 18-51 years) underwent open rhinoplasty with augmentation. Moreover, over half (53.85%) had prior rhinoplasty with implants that caused skin thinning.

In this surgical technique, costal cartilage was used to create a dorsal onlay graft (placed on top of the tissue). A modified technique involved covering only the edges of the graft with perichondrium, the authors rationalized this modification by that the traditional methods (covering the entire graft) were avoided due to potential swelling and unnatural look.

The authors measured the outcomes via the Rhinoplasty Outcomes Evaluation (ROE) scale, scores for each item (e.g., tip definition, projection) and they reported that the total score significantly improved after surgery (p < 0.001), indicating patient satisfaction with aesthetics and function. They also used the VAS questionnaire, and patients reported increased satisfaction with the nasal dorsum post-surgery (pre-operative: 2.45 ± 1.34, post-operative: 8.55 ± 0.87). The follow-up period was between six and 20 months (limited for long-term assessment). There were minimal complications (fat liquefaction, graft warping requiring revision), but no graft extrusion or infections were reported.

The study suggests that covering only the edges of the dorsal graft with perichondrium might be a promising technique for rhinoplasty in thin-skinned patients. It resulted in high patient satisfaction and minimal complications. However, due to the study design limitations (small sample size, no control group, short follow-up), more robust studies are needed for conclusive evidence.

Level of Evidence IV: A Prospective Study With Limitations

Karaaltin et al. investigated the use of fascia lata grafts harvested from the thigh to improve cosmetic outcomes in rhinoplasty patients with thin skin [12]. 63 patients (26 males, 37 females) underwent rhinoplasty between May 2004 and December 2005. Their age ranged from 18 to 43 years (average 28.7 years). All patients had thin nasal skin and 49 were primary rhinoplasty cases, 14 were secondary (revision) surgeries.

The authors employed a fascia lata graft harvested from the right lateral thigh (average size 2 x 3 cm) that was placed over the nasal dorsum (bridge) to camouflage irregularities then sutures secured the graft in position. During the 14-26 months follow-up period, questionnaire results showed that all patients were satisfied with the cosmetic results with minimal donor site concerns. There were no visible irregularities observed on the nasal dorsum post-surgery. Only one patient required revision surgery due to a separate accident, and the fascia lata graft appeared healthy and in place. One patient reported a scar and pain at the thigh donor site (which was treated with a compression bandage).

The study suggests fascia lata grafts might be a viable option for covering grafts used in rhinoplasty on patients with thin skin. It resulted in high patient satisfaction and minimal complications. However, due to the study design limitations (no control group).

Level of Evidence IV: A Prospective Study With Limitations

Cardenas et al. investigated lipoinjection of microfat grafts to improve rhinoplasty outcomes, specifically targeting patients with thin skin [13]. The study included 78 Colombian patients (71 women, seven men) who underwent rhinoplasty between February 2003 and May 2005 with a range age between 14 and 56 years. The patients underwent both primary (61) and secondary (17) rhinoplasty.

The fat was harvested from the inner knee using a small cannula and then injected throughout the nose (radix, dorsum sides, supratip) and over the bony framework. The amount of fat injected varied based on individual needs. The follow-up period was between one and 36 months.

The authors reported that 77 patients were satisfied with the results. One patient reported dissatisfaction unrelated to lipoinjection as declared by the authors. Furthermore, no major complications were reported (infection, hematoma, bleeding). No irregularities were observed, and skin quality improved in all patients.

Level of Evidence IV: A Prospective Study With Limitations

Lozada et al. reported the results of a prospective study on the use of supracrural ligament graft in rhinoplasty [14]. The study enrolled 49 patients undergoing primary rhinoplasty. All surgeries were performed by the senior author using an open rhinoplasty approach.

In this surgical technique, the supracrural ligament graft (the average graft sized 0.6*0.4 cm) was harvested during the initial exposure of the nose. It was then used to camouflage or augment irregularities in different areas of the nose including the radix, dorsum, and tip.

The study found that the supracrural ligament graft was a safe and effective technique for obtaining graft material during rhinoplasty. The authors stated that it eliminated the need for an additional harvest site and provided a soft tissue graft for addressing contour irregularities. No complications were reported in any of the patients. However, the follow-up period was not mentioned in the study.

Overall, the supracrural ligament graft appeared to be a promising technique for obtaining graft material during rhinoplasty. However, more research is needed to confirm its long-term safety and efficacy.

Level of Evidence IV: A Prospective Study With Limitations

Mohebbi et al. reported their randomized controlled trial comparing two types of grafts used in rhinoplasty surgery for thin-skinned patients [15]. Acellular dermis (ACD) and fascia lata were used to cover the dorsum of the nose after hump reduction.

A total of 68 patients underwent rhinoplasty between 2013 and 2015. 42 patients completed the 12-month follow-up (24 in the ACD group and 18 in the fascia lata group). Their ages ranged from 22 to 55 years old.

After removing the dorsal hump, either ACD or fascia lata was placed over the dorsum of the nose. Fascia lata grafts resulted in less dorsal irregularity as detected by feel (palpation) at 12 months compared to ACD grafts(P=0.001). Patients were also more satisfied with the results of fascia lata grafts. ACD grafts showed a higher absorption rate than fascia lata grafts at 6 months (P= 0.04), but this difference disappeared by 12 months (P= 0.386).

Overall, this study suggests Fascia Lata grafts may lead to better long-term outcomes in terms of feeling (palpation) and patient satisfaction compared to ACD grafts in rhinoplasty for thin-skinned patients. However, more research might be needed to confirm these findings in a larger, blinded study.

Level of Evidence I: Randomized Controlled Trial

Bertossi et al. performed a prospective study investigating a new surgical technique for rhinoplasty [16]. The goal was to improve the long-term outcome, especially for thin-skinned patients, by preventing irregularities on the nasal dorsum. Fifty-seven patients (26 males, 31 females) aged 31-55 years underwent rhinoplasty between 2006 and 2010. Patients were divided into three groups based on the size of the nasal dorsum defect they had (less than 1mm, 1-2mm, or greater than 3mm).

An open rhinoplasty approach was used. Crushed septal cartilage was wrapped in a collagen membrane to create a graft. The thickness of the graft depended on the size of the defect. The graft was placed over the nasal dorsum after corrective surgery to address the defect.

All patients experienced an initial over-correction that resolved within 14-30 days. Biopsy results at six months showed the membrane was replaced by collagen fibers. The long-term volume reabsorption was about 5%. There was one case of dislocation of the graft due to patient manipulation. Five patients required minor touch-up procedures at the supratip area.

Regarding the complications, no infections or skin color modifications were reported. The pain was mild except for one patient who experienced moderate pain.

The study suggested that bilayered combined cartilage and collagen membrane grafts may offer several advantages in rhinoplasty, particularly for thin-skinned patients. The bilayered graft may lead to superior aesthetic outcomes with a balanced nasal tip and a fuller dorsum. The technique avoids altering the thickness and texture of the overlying skin. This is especially important for thin-skinned patients who are more prone to showing irregularities through the skin. By using a collagen membrane (Bio-guide), the need for an additional cartilage harvesting site is eliminated. This can potentially shorten surgery time and reduce donor site morbidity.

Level of Evidence IV: A Prospective Study With No Control Group

Ozturan et al. investigated the use of a novel surgical technique in rhinoplasty to address potential dorsal irregularities [17]. The technique involved harvesting a ligament (dermocartilaginous ligament) from the tip of the nose and using it as a flap to cover the nasal dorsum. Eleven patients (six males, five females) with an average age of 26 underwent primary rhinoplasty. Skin thickness was assessed by manual palpation (eight patients had thin, two had medium and one patient had thick skin).

For the surgery, an external rhinoplasty approach was used. The dermocartilaginous ligament was dissected and converted into a thin flap. The flap was then stretched and draped over the nasal dorsum to camouflage any minor irregularities. All patients were followed for an average of 27 months. No patients developed infections or hematomas. There were no visible irregularities on the nasal dorsum post-operatively and all patients were satisfied with the aesthetic outcome.

Level of Evidence IV: A Retrospective Study With No Control Group

Another study by Bertossi et al. investigated the feasibility and outcomes of laser-assisted rhinoplasty compared to the traditional open rhinoplasty technique [18]. Fifty patients between 18 and 51 years old undergoing primary rhinoplasty were enrolled and randomly assigned to either laser-assisted rhinoplasty (using erbium: yttrium-aluminum-garnet laser emitting at a wavelength of 29.4 mm) or classic open rhinoplasty. All surgeries were performed by the same senior author and a standard transcolumellar open approach was used for both groups.

In the laser-assisted rhinoplasty group, the erbium: yttrium-aluminum-garnet laser was used to perform osteotomies, resection of the crura and nasal hump. In the traditional rhinoplasty group, a standard transcolumellar open approach was used.

 At 12-month follow-up, patients in the laser-assisted rhinoplasty group showed a lower rate of residual defect (0%) compared to the traditional rhinoplasty group (12%). Additionally, patients in the laser-assisted rhinoplasty group reported higher satisfaction rates (96% vs 80%) and experienced less bleeding, edema postoperatively, and a faster recovery time. No major complications were observed in either group. However, the laser surgery did take slightly longer to perform than the traditional surgery (115 Vs 103 minutes, respectively). The follow-up period was 24-36 months.

Level of Evidence IV: A Retrospective Study With No Control Group

Kovacevic et al. explored the potential of a new technique in rhinoplasty to address concerns about thin or damaged skin [19]. In a series of 107 cases, surgeons investigated diced macrofat transfer combined with PRF for nasal augmentation. Fat was harvested from either the abdomen (costal region) or the belly button (umbilical region) of the patients themselves. This harvested fat was then meticulously chopped into small pieces (diced). PRF, derived from the patient's own blood (to promote healing and tissue growth), was prepared using a centrifugation process. Finally, the diced fat was combined with the PRF to create a composite graft, then transplanted to the nasal dorsum, the bridge of the nose.

The researchers monitored the patients for an average of 14 months after surgery revealing good overall skin quality, there were no signs of shrinkage, scarring, or discoloration of the skin. Moreover, imaging techniques confirmed that the transferred fat grafts had survived. Importantly, no complications or issues with wound healing were reported during the perioperative period (around the time of surgery). While these findings are encouraging, more robust research, such as controlled trials, would be needed to confirm these observations.

Level of Evidence IV: A Prospective Study With No Control Group

Storck et al. emphasized the work by Kovacevic as they investigated the use of autologous fat transfer with PRF in septophinoplasty (SRP) procedures [19,20]. They conducted a prospective case series involving 21 patients who underwent SRP and had either thin skin or scarring from previous procedures. The surgical technique involved harvesting fat from the periumbilical or rib region, mincing and purifying it, and then mixing it with PRF to create a graft. This graft was then transferred to the nasal dorsum to improve the quality of the soft tissue envelope and conceal irregularities. The patients were followed up for 1 year after surgery. Both sonography and magnetic resonance imaging (MRI) confirmed the survival of the fat grafts in all patients. Additionally, in vitro analysis revealed variations in the quality and quantity of transplanted fat cells between individuals. Overall, the study suggested that autologous fat transfer with PRF may be a beneficial technique for improving the cosmetic outcome of SRP in patients with thin skin or scarring. However, it is important to note that this is a low-level evidence study (case series), and further research is needed to confirm these findings and assess patient satisfaction and long-term side effects.

Level of Evidence IV: A Prospective Study With No Control Group

A study conducted by Niechajev et al. included 44 patients (29 females and 15 males) who underwent surgery between 1997 and 2010 to evaluate the Skoog rhinoplasty technique for thin-skinned patients [21]. The patients ranged in age from 18 to 47 years (average 27 years). Of the patients, 61% were of Middle Eastern descent. An endonasal technique was used in all but four cases. 

The study found favorable outcomes in all cases. The Skoog technique effectively created a smooth nasal bridge and prevented irregularities that can occur with traditional reduction rhinoplasty. Additionally, it served as a useful rescue technique when too much dorsum was removed during surgery. No patients experienced visible resorption of graft, infection, or delayed healing. Reinsertion of the hump prolonged the operation time by 7-10 minutes. In some cases (n=4), a thin plate-like graft resulted in visible and palpable edges, which necessitated secondary shaving or filing.

Level of Evidence IV: A Prospective Study With No Control Group

Baptista et al. investigated the use of lipofilling (fat grafting) to correct complications following rhinoplasty surgery [22]. The study included 20 women (average age 53) who underwent lipofilling between 2006 and 2012 to address rhinoplasty sequelae in thin-skinned patients.

Two techniques were used. Initially, surgeons used the Coleman technique with general anesthesia and 11-gauge cannulae. Later, they developed a microinjection technique with local anesthesia and 21-gauge cannulae. In both techniques, fat was harvested from the abdomen or knees, processed, and injected into the nasal region. The amount of fat injected ranged from 1 to 6 cc.

Regarding the outcomes, eighteen patients achieved satisfactory results after one procedure. Two patients required a second lipofilling session. Complications were minimal, with some patients experiencing bruising and swelling after the Coleman technique procedure. The microinjection technique resulted in less bruising and swelling.

Eighteen patients were satisfied or very satisfied with the results. Two patients required a second fat grafting session due to insufficient correction. Surgeons also rated the outcomes as very good in 14 cases and good in three cases at the 18-month follow-up. Bruising and swelling were the main side effects, more common with the Coleman technique.

Discussion

This review analyzed various studies investigating techniques for addressing dorsal deformities in rhinoplasty for patients with thin skin. The studies explored various graft materials and surgical techniques to achieve optimal cosmetic outcomes while minimizing complications.

Santos et al. showed that SC+PRF is more effective for dorsal camouflage compared to DC alone in patients with thin skin [8]. PRP contains growth factors that can promote healing, cell survival, and tissue regeneration. This might contribute to better graft integration and reduced resorption of the DC [8]. The PRP may act as a scaffold or "glue" to hold the DC fragments together, potentially leading to a smoother and more stable dorsal contour, particularly relevant for thin skin where irregularities are more noticeable [23].

The use of DC with NCH gel and blood for dorsal camouflage in rhinoplasty shows promise as a minimally invasive technique. DC offers a natural material for camouflage, potentially blending better with surrounding tissues compared to synthetic implants [9]. The NCH gel's high viscosity might facilitate easier application of the DC mixture and potentially reduce the risk of postoperative irregularities. Furthermore, NCH gel may help decrease scar tissue formation at the surgical site, potentially contributing to a smoother long-term outcome [24]. This method showed positive results in a small sample size, requiring further investigation with larger studies.

Several factors might contribute to the superiority of fascia lata grafts in thin-skin rhinoplasty compared to ACD grafts as reported by Mohebbi et al. RCT [15]. This could be contributed to that fascia lata is a stronger and more durable tissue compared to ACD, potentially offering better long-term support and reducing the risk of irregularities, integrating more seamlessly with surrounding tissues, leading to a smoother feel on palpation, furthermore, ACD grafts can sometimes contract over time, potentially causing irregularities on the nasal dorsum. Fascia lata may be less prone to this complication. Karaaltin et al. also supported that fascia lata grafts were associated with high patient satisfaction and minimal complications [12]. However, the absence of a control group limits the strength of the later evidence.

While traditional graft materials like cartilage remain prevalent in rhinoplasty, several studies explored alternative graft materials with promising results. Lozada et al. investigated the supracrural ligament graft [14], while Ozturan et al. utilized a dermocartilaginous ligament flap from the tip of the nose [17]. Both grafts are harvested from the patient's own nose, potentially offering a better tissue match, these ligamentous structures may provide a natural layer of soft tissue coverage over traditional grafts (cartilage, etc.), potentially leading to a smoother and more natural-looking nasal dorsum. It also avoids the need for additional surgical sites to harvest donor material, leading to less overall surgical trauma and faster recovery. However, further research is necessary to establish these techniques as definitive solutions.

Cardenas et al. and Kovacevic et al. showed encouraging results with fat grafting techniques using microfat and diced macrofat combined with PRF for nasal augmentation in patients with thin skin [13,19]. Microfat may have a higher engraftment rate due to its increased surface area for vascularization [25,26]. Diced macrofat can provide better structural support and volume restoration [27]. Moreover, PRF contains growth factors that can promote fat cell survival and improve blood vessel formation within the graft [28]. Despite these encouraging initial results, both studies involved a limited number of participants and more robust studies are needed to confirm these findings.

Lasers offer a more precise tool for tissue manipulation compared to traditional surgical instruments. This increased precision can be particularly advantageous for thin-skinned patients as it minimizes unnecessary tissue disruption and manipulation, lasers can also reduce bleeding, which promotes better vision and edema and quicker recovery [18]. However, it is important to note that laser-assisted rhinoplasty is a relatively new technique, and more long-term studies are needed to confirm its advantages and potential risks.

Niechajev et al. argued that the Skoog method of rhinoplasty remains a valuable option to consider and use when appropriate [21,29], as when too much of the dorsum height is eliminated, the option of reinserting the excised hump is a suitable rescue strategy preventing the parrot peak deformity.

Baptista et al. endorsed that minor nasal dorsum and tip shape defects can be fixed with lipofilling, supported later by Challita [22,30]. Since lipofilling is less intrusive, patients with thin skin, who are more prone to have noticeable scars following surgery, will recover from the procedure more quickly and leave with less visible scars [31]. Lipofilling does have certain drawbacks, though, such as a high rate of resorption, unpredictable results, and a limited variety of uses [25].

Additional considerations

Careful assessment of skin quality and patient expectations is crucial. The success of these techniques relies heavily on the surgeon's skill and experience. More extended follow-up periods are needed to assess the durability of the results. Choosing the optimal technique for a particular patient requires a comprehensive discussion between the patient and a qualified rhinoplasty surgeon, considering individual factors and desired outcomes.

Limitations

Many studies included in this review are Level IV with limitations such as small sample sizes, no control groups, and short follow-up periods. More high-quality studies (Levels I and II) are needed to confirm the efficacy and safety of various techniques for thin skin rhinoplasty.

Future directions

Well-designed studies comparing different graft materials and techniques are necessary. Long-term follow-up data are crucial to assess the sustainability of aesthetic outcomes. Patient satisfaction surveys should be incorporated to evaluate subjective experiences.

## Conclusions

Several techniques show promise for addressing dorsal deformities in rhinoplasty for thin-skinned patients. DC with PRF, fascia lata grafts, and laser-assisted rhinoplasty appear to be particularly effective based on the available evidence. However, more high-quality studies with larger sample sizes and control groups are necessary to confirm these findings and establish long-term safety and efficacy.

## Appendices

**Table 2:** Search performed on articles published in English

Search terms	Library	Number of retrieved articles		Number of articles	Causes for exclusion
((rhinoplasty) AND ((thin) AND (skin)))	PubMed	135	included after removal of duplications	776	Automatic duplicate removal
	Science direct	570	included after abstract screening	49	
	WOS	124	included after full text screening i.e. to extraction	15	Review 5 Non-English study 1 Irrelevant 18 Not addressing thin skin clearly 8 Duplicates 2
	Scopus	128			
	Central	8			
	total	965			
